# *SMAD5* as a novel gene for familial pulmonary arterial hypertension

**DOI:** 10.1042/CS20241340

**Published:** 2025-01-15

**Authors:** Ding Cao, Ekkehard Grünig, Yuriy Sirenko, Ganna Radchenko, Henning Gall, Ayat Ahmed, Susanne Theiß, Mareike Lankeit, Benjamin Meder, Magdalena Laugsch, Christina A. Eichstaedt

**Affiliations:** 1 Center for Pulmonary Hypertension, Thoraxklinik Heidelberg gGmbH at Heidelberg University Hospital, Translational Lung Research Center Heidelberg (TLRC), German Center for Lung Research (DZL), Heidelberg, Germany; 2 Institute of Human Genetics, Heidelberg University, Heidelberg, Germany; 3 Pulmonary Hypertension Center, SI NSC named after M.D. Starzhesko of NAMS of Ukraine, Kyiv, Ukraine; 4 Department of Pneumology, Medical and Policlinic II, University Hospital of Giessen and Marburg, Universities of Giessen and Marburg Lung Center (UGMLC), German Center for Lung Research (DZL), Giessen, Germany; 5 Center for Thrombosis and Hemostasis, University Medical Center Mainz, Mainz, Germany; 6 Department of Internal Medicine III, Precision Digital Health, University of Heidelberg and Informatics for Life and German Center for Cardiovascular Research (DZHK), Heidelberg, Germany

**Keywords:** BMPR2 pathway, genetics, pulmonary arterial hypertension

## Abstract

Genetic diagnostic testing of 325 pulmonary arterial hypertension (PAH) patients using a PAH specific gene panel including 18 known PAH genes revealed mutations in 23%. Further PAH candidate genes were sequenced in the remaining patients exposing two *SMAD5* variants, which were clinically and functionally characterized. We first recorded familial cosegregation and clinical parameters. Functional tests were performed following transient over-expression of the two *SMAD5* variants in pulmonary artery smooth muscle cells (PASMCs). Expression of these variants was confirmed by quantitative PCR, Sanger sequencing, and Western blotting. Cell viability was evaluated using cell counting kit 8, cell proliferation by bromodeoxyuridine (BrdU), and apoptosis by annexin V assay. Both *SMAD5* missense variants were absent in healthy controls and predicted to be pathogenic. The variant c.1175T>C p.(Leu392Pro) was identified in a heritable PAH patient and her healthy son. The mother had died of suspected PAH at age 42. The expression of this variant in PASMCs led to significantly higher cell viability due to higher proliferation in comparison with *SMAD5* wild-type cells. The second variant c.277T>A p.(Trp93Arg) was identified in a patient with congenital heart disease associated PAH with a surgically repaired ventricular septal defect. Its expression led to significantly lower cell viability due to increased apoptosis in comparison with wild-type *SMAD5* cells. Taking into account familial aggregation, clinical findings, and functional evidence, both variants could be classified as likely pathogenic. This is the first description of *SMAD5* as a potential novel PAH gene for genetic diagnostic testing.

## Introduction

Pulmonary arterial hypertension (PAH) is a rare disease characterized by elevated pulmonary vascular resistance, right ventricular hypertrophy, and ultimately right heart failure [1]. Genetic mutations in up to 18 known PAH genes can lead to abnormal proliferation and apoptosis of pulmonary vascular smooth muscle cells, endothelial cells, and fibroblasts resulting in thickening of the pulmonary arterial walls [2]. In familial PAH patients 85% and ~15% of idiopathic PAH cases present with a disease-causing genetic mutation [3]. The main causative gene is the bone morphogenetic protein receptor type 2 (*BMPR2*), which was first described as disease-causing for PAH in the year 2000 [4,5] and is part of the transforming growth factor beta (TGF-β) super family. Since its discovery, further genes within and outside the BMPR2 pathway have been described as causative for PAH [6]. BMPR2 signaling is mediated after binding of bone morphogenetic proteins to BMPR2 and a type 1 receptor via ‘mother against decapentaplegic homologue’ (SMAD) proteins. SMAD5, SMAD8, and SMAD1 each form a complex with the ‘common’ SMAD protein, SMAD4 [7]. All SMAD proteins carry a DNA-binding MAD homology 1 domain (MH1) and a protein binding MH2 domain [8].

This transcriptionally active SMAD heterotrimer of, e.g. two SMAD5 proteins and one SMAD4 protein, binds to gene promoters and enhancers in the nucleus. As a consequence, the SMAD5 complex activates or inhibits genes, which are essential for embryonic development, cell differentiation, angiogenesis, and tissue homeostasis in an anti-proliferative and pro-apoptotic manner [9–11]. In PAH patients, BMPR2 and subsequent SMAD signaling are dysregulated leading to vessel remodeling [12].

Currently, in Heidelberg, 18 PAH genes are routinely sequenced by next-generation sequencing using our patented PAH gene panel (EP3507380) at the largest German referral center for genetic testing of PAH patients [3]. By this approach, we previously identified mutations in 23% of 325 consecutive PAH patients [3]. However, in the same study, there were still families with heritable PAH (HPAH) and patients with other possibly genetically determined forms of PAH such as PAH associated with congenital heart disease (CHD-APAH) without a genetic cause in any of the currently known PAH genes. In these patients, we sequenced up to 41 further candidate genes including *SMAD5*, which has not been identified before as disease-causing gene for PAH.

In the cohort of the remaining 251 PAH patients without a disease-causing variant in any of the 18 known PAH genes, we detected two rare and novel missense variants in *SMAD5*. The variant c.1175T>C p.(Leu392Pro) was identified in a HPAH family and the variant c.277T>A p.(Trp93Arg) in a CHD-APAH patient. In the present study, we therefore aimed to clinically characterize the variant carriers and functionally assess the role of the two novel *SMAD5* variants in cell proliferation, apoptosis, and viability *in vitro*.

## Materials and methods

### Clinical characterization of subjects and variant classification

Patients had a confirmed PAH diagnosis following the diagnostic algorithm and invasive right heart catheterization as outlined in the current guidelines [13]. Family and medical history were obtained; physical examination, echocardiography, World Health Organization (WHO) functional class, 6-min walking distance, and laboratory parameters were measured including N-terminal pro-brain natriuretic peptide (NT-proBNP) as described previously [3,14].

From a previously reported cohort of 325 PAH patients [3], the 251 patients without any disease-causing variant within the 18 known PAH genes (*ABCC8, ACVRL1, AQP1, ATP13A3, BMPR1B, BMPR2, CAV1, EIF2AK4, ENG, GDF2, KCNA5, KCNK3, KDR, KLF2, SMAD4, SMAD9, SOX17, TBX4*) received further genetic testing in up to 41 other research genes (*ACVR1, BMP2, BMP7, BMPR1A, BTNL2, COX4I2, COX5A, CREB1, CYP1B1, EPAS1, FLNA, FOXF1, FOXO1, GGCX, GUCY1A3, HRG, ID1, ID2, ID3, ID4, IL6, JAK2, KLF4, KLF5, KLK1, NOTCH3, PHF14, RASA1, SMAD1, SMAD5, SMAD6, SMAD7, SMYD2, SOD2, TBX2, THBS1, TMEM70, TOPBP1, VCAN, VHL, ZFYVE16*). Coding sequences and exon-intron boundaries were included. This resulted in the two *SMAD5* missense variants, which were classified using the variant interpretation guidelines from the American College of Medical Genetics and Genomics (ACMG) [15]. The *in silico* prediction program rare exome variant ensemble learner (REVEL) was used to assess pathogenicity of missense variants. The program combines the scores of 13 tools into a single prediction. Scores 0.75–1.0 were considered pathogenic [16].

Pathogenic and likely pathogenic variants are referred to as ‘mutations’ in the present study.

All study participants provided written informed consent for further genetic research testing. The study complied with the Declaration of Helsinki in its current version.

### Cell culture

Primary, commercially available, pulmonary artery smooth muscle cells (PASMCs) from a deceased 28-year-old, Asian male donor without alcohol, smoking, or drug history and who died of anoxia (C0095C, lot-ID 1809196, ThermoFisher, U.S.A.) were cultured for the majority of experiments with medium 231 (M231500, ThermoFisher, U.S.A.) and smooth muscle growth supplement (S00725, ThermoFisher, U.S.A.) for passaging and downstream functional analyses. A second lot of PASMCs (C0095C, lot-ID 1792537, ThermoFisher, U.S.A.) from a 45-year-old African American male donor with an unknown cause of death was used for initial experiments. For cell differentiation, PASMCs were cultured with medium 231 plus smooth muscle differentiation supplement (S0085, ThermoFisher, U.S.A.). Trypsin (R001100, ThermoFisher, U.S.A.) was used for passaging cells. All experiments used PASMCs from passages 4 to 6.

### Immunofluorescence staining

Characteristic alpha-smooth muscle actin (α-SMA) expression in differentiated PASMCs (lot 1792537) was confirmed by immunofluorescence (Supplementary Figure S1). Fixed and permeabilized PASMCs were incubated with a primary antibody against α-SMA (A5228-25UL, sigma, U.S.A.; dilution 1:100) at 4°C overnight. Following three washes with phosphate-buffered saline (PBS), samples were incubated with a fluorescently labeled Alexa Fluor™ 488 secondary antibody (A11029, ThermoFisher, U.S.A.) at a 1:500 dilution at room temperature for 1 h. Nuclei were counterstained with DAPI (62248, ThermoFisher, U.S.A.), with a 1:1000 dilution for 5 min. After a final PBS wash, samples were mounted using an anti-fade mounting medium (0100–01, Southernbiotech, U.S.A.) and cover slips.

### siRNA transfection

Short interfering RNA (siRNA) was used (silencer select siRNA, sc-38378, Santa Cruz, U.S.A.) to target *SMAD5* and compared with an equivalent amount of unspecifically binding scramble control siRNA (sc-37007, Santa Cruz, U.S.A.). A total of 1 × 10^5^ PASMCs (lot 1809196) were cultured one day before transfection in a 12-well plate. For transfection, 3 µl of siSMAD5 and siControl were combined with 1.875 µl of Lipofectamine 3000 (L3000001, ThermoFisher, U.S.A.). Protein extraction was performed 48 h after transfection.

### Plasmid transfection

The pCMV6-SMAD5-myc-DDK plasmid with a kanamycin and neomycin resistance gene (RC201949, ORIGENE, Germany) was used to express *SMAD5* (transcript: ENST00000545279, reference sequence: NM_005903). The pCMV6-myc-DDK plasmid, kindly provided by Miss. Hipp from the Department of Human Genetics at Heidelberg University, Germany, served as a transfection control. A total of 1 × 10^5^ PASMCs (lots 1809196 and 1792537) were cultured one day in a 12-well plate before transfection. For transfection, 2 µl of Lipofectamine LTX (15338500, ThermoFisher, U.S.A.) and 0.5 µl of CombiMag (CM20200, OZ Biosciences, France) were combined with 0.5 µg of plasmid and introduced into the PASMCs by placing the cell culture plate on the magnetic plate (MF10000, OZ Biosciences, France) for 30 min (magnetofection). RNA extraction was carried out 24 h post-transfection, while protein extraction was done after 48 h.

### Site-directed mutagenesis

Site-directed mutagenesis was performed using the QuikChange II XL Site-Directed Mutagenesis Kit (200523, Agilent Technologies, U.S.A.) according to the manufacturer’s instructions. A plasmid containing the *SMAD5* wild-type (wt) gene was used as the template. Primers containing the desired mutation were designed using the QuikChange Primer Design tool (Agilent Technologies): *SMAD5_*p.L392P_fwd (ggagtttgctcagcttccggctcaatctgtcaacc), *SMAD5_*p.L392P_rev (ggttgacagattgagccggaagctgagcaaactcc), *SMAD5*_p.W93R_fwd (cccatgttatatattgtcgtgttaggcgctggccgg), and *SMAD5*_p.W93R_rev (ccggccagcgcctaacacgacaatatataacatggg). PCR included initial denaturation at 95°C for 1 min, followed by 18 cycles of denaturation at 95°C for 50 s, annealing at 60°C for 50 s, and extension at 68°C for 17:30 min with a final extension of 7 min. The reaction mixture was treated with *Dpn* I to digest the methylated parental DNA. The mutated plasmids were transformed by heat shock bacteria transformation protocol into competent TOP 10 *Escherichia coli* bacteria and amplified within. MiniPrep (A1223, Promega, U.S.A.) was used to extract mutant plasmid at high concentrations.

### Sanger sequencing

PCR amplification of the target region was carried out using the Phusion High-Fidelity PCR Kit (F553L, ThermoFisher, U.S.A.) with custom-designed gene-specific primers *SMAD5_fwd* ccaccagcccaacaacactcc and *SMAD5_rev* gctgggagctgaaatggacttcc. Co-segregation was performed using the primers SMAD5-X7-F-M13 gtaaaacgacggccagtgaatgcctcagtgacagcag and SMAD5-X7-R-P172 tatagggcgaattgggtcagcccctattcaatatcaaaatc. The PCR program included initial denaturation at 94°C for 2 min, followed by 35 cycles of denaturation at 94°C for 30 s, annealing at 55°C for 30 s, extension at 72°C for 1:30 min and a finalextension at 72°C for 5 min. The PCR product was sent to Microsynth, Germany, for sequencing.

### Western blotting

Total protein was extracted from cells with radio-immuno-precipitation buffer (89900, ThermoFisher, U.S.A.) supplemented with protease and phosphatase inhibitor cocktail (78420, ThermoFisher, U.S.A.). For immunoblotting total anti-SMAD5 antibodies (ab40771, Abcam, U.K.; dilution 1:2500) and glyceraldehyde 3-phosphate dehydrogenase (GAPDH, ab8245, Abcam, U.K.; dilution 1:2500) were added. Anti-rabbit secondary antibody was used for SMAD5 (1706515, BIO-RAD, U.S.A.; dilution 1:2500) and anti-mouse secondary antibody (1706516, BIO-RAD, U.S.A.; dilution 1:2500) for GAPDH.

### Real-time PCR

RNA extraction was performed 24 h after plasmid transfection (InnuPREP RNA Mini Kit 2.0, 845-KS-2040050, Analytik Jena, Germany). The first chain of copy DNA (cDNA) was created by First Strand cDNA Synthesis Kit (E6560L, NEB, U.S.A.), and messenger ribonucleic acid (mRNA) was measured by quantitative polymerase chain reaction (qPCR) (PB20.11–05, PCRBiosystems, U.K.). The forward primer (tctccaaacagcccttatccc) was bound in exon 2 of *SMAD5* and the reverse primer 8 (gcaggaggaggcgtatcag) in exon 3. *GAPDH* was used as a housekeeping reference gene (*GAPDH*_fwd: gcaccgtcaaggctgagaac *GAPDH*_rev agggatctcgctcctggaa), and cycle threshold (CT) values between *SMAD5* and *GAPDH* were calculated by 2^−ΔΔCT^ [17].

### Apoptosis, proliferation, and cell viability assays

Per well 1 × 10^4^ PASMCs (lots 1809196 and 1792537) were cultured on a 96 well-plate one day prior to transfection. Transfections were performed using 0.4 µl of Lipofectamine LTX (15338030, ThermoFisher Scientific, U.S.A.) and 0.1 µl of CombiMag (CM20200, OZ Biosciences, France) combined with 0.1 µg of plasmid. All measurements and analyses followed the manufacturers’ instructions. Apoptosis was measured on the Incucyte S3 Live Cell Analysis System with the Incucyte Annexin V Red Dye (4641, SARTORIUS, Germany). The dye was diluted with cell culture medium 1:200, and results were normalized to cell confluency.

For proliferation, BrdU (ab126556, Abcam, U.K.) reagent was added to the PASMCs at 24 h, 48 h, or 72 h post-transfection. Optical density (OD) was measured at 450 nm and 550 nm using a multimode-microplate reader (Spark, Tecan, Switzerland).

For the cell viability, measurement cck-8 (ab228554, Abcam, U.K.) reagent was added at a 1:10 dilution to the PASMCs (lots 1809196 and 1792537) at 24 h, 48 h, or 72 h post-transfection. The cells were then incubated for 3 h, and OD was measured at 460 nm using the same microplate reader. The cell viability or cell proliferation rate was calculated using the following formula: (experimental OD value − blank OD value) / (control OD value − blank OD value) × 100%.

### Inhibitor treatment

LDN193189 (ALK1/2/3/6 inhibitor, SML0559, Sigma-Aldrich, U.S.A.) was diluted to 5 nm, 50 nm, and 500 nm. Concentrations were previously shown to inhibit SMAD signaling in murine mesenchymal precursor cells [18]. SB431542 (ALK4/5/7 inhibitor, S4317-5mg, Sigma-Aldrich, U.S.A.) was diluted to 1 µm, 5 µm, and 50 µm [19]. For the wt (non-transfection) PASMC (lot 1809196) treatment, 5 nm, 50 nm, and 500 nm of LDN193189 or 1 µm, 5 µm, and 50 µm of SB431542 were added 24 h after transfection and incubated for 24 h. The cell medium without LDN193189 or SB431542 was used as the negative control treatment.

### Statistical analysis

All measurements and analyses were performed as biological triplicates. All statistical analyses were performed using Prism 10 software (GraphPad). A *P* value < 0.05 was considered significant. Statistical comparisons between two groups for *in vitro* studies were performed using an unpaired t-test. Comparison among three or more groups was performed using one-way analysis of variance followed by a Dunnet analysis. Experiments were carried out as triplicates. Parameters are displayed as mean and standard deviation.

## Results

### HPAH family carried *SMAD5* missense variant c.1175T>C p.(Leu392Pro)

**Figure 1:**
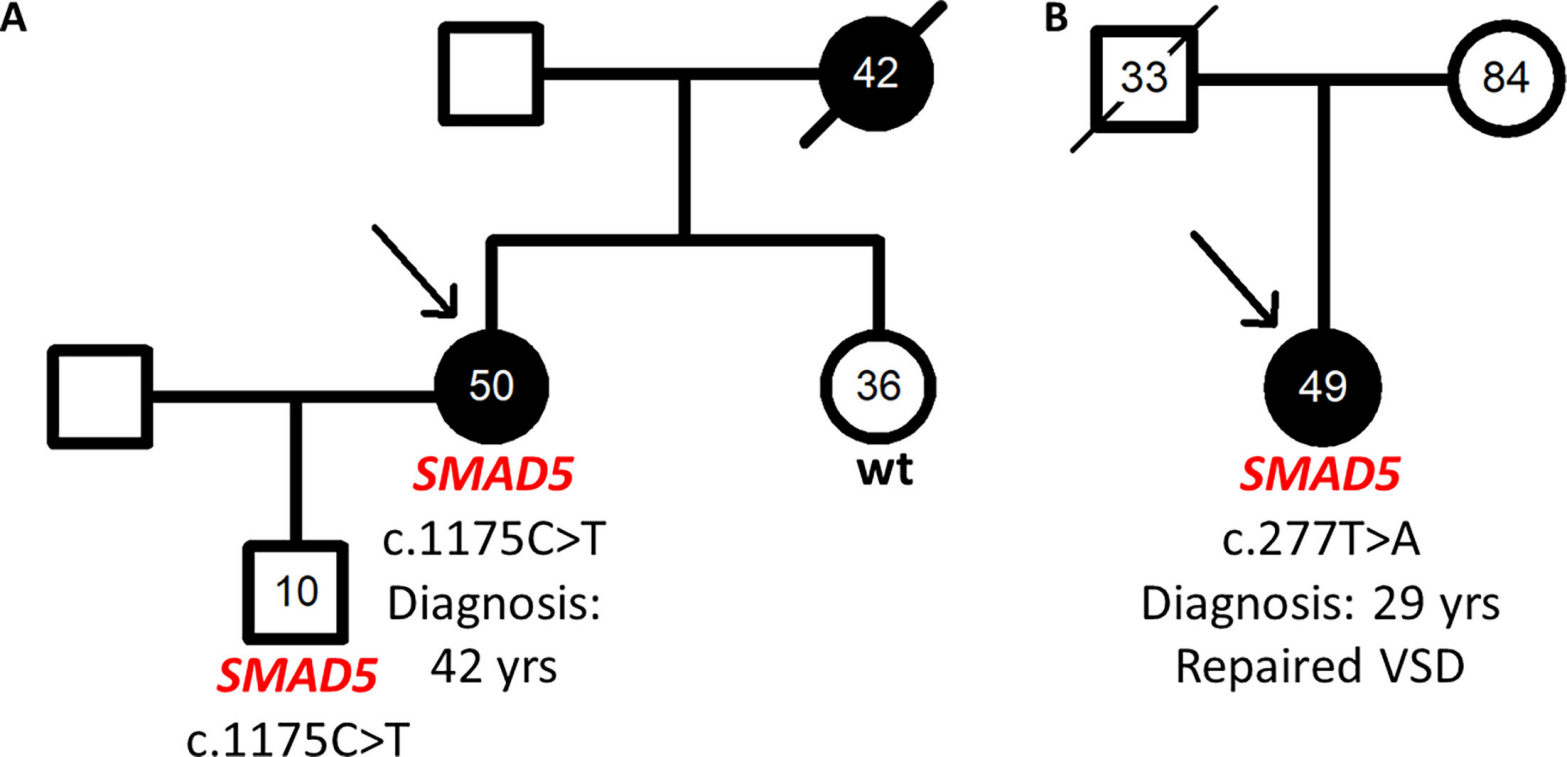
Pedigrees of the *SMAD5* families. **(A**) The index patient was diagnosed eight years ago aged 42 years. According to the index patient, her mother had died of severe PAH aged 42 years. The clinical values were not available to us. The index patient carried the *SMAD5* variant c.1175T>C p.(Leu392Pro) as did her healthy son. The index’s healthy sister had the wild type (wt) of *SMAD5*. (**B**) The index patient was diagnosed 20 years ago aged 29 years and carried the *SMAD5* c.277T>A p.(Trp93Arg) variant. Her father had a ventricular septal defect (VSD) and died aged 33 years of an unrecorded cause. Circles denote females, squares males, strike through deceased individuals, and filled symbols pulmonary arterial hypertension.

The first *SMAD5* missense variant was identified in a Ukrainian HPAH family (Figure 1). In the patient’s family, the currently 50-year-old female index patient was diagnosed with HPAH aged 42 years after 2.5 years of preceding dyspnea (Figure 1A). The index’s mother had died of suspected PAH aged 42 years, and the index’s father was unavailable for testing due to the Ukrainian war. The *SMAD5* variant was also present in her to-date healthy 10-year-old son. The 36-year-old index patient’s sister was healthy and had no mutation (*SMAD5* wt, Figure 1A). Both son and sister had a normal echocardiogram with no signs of PAH. The index patient displayed a severe PAH with a mean pulmonary arterial pressure (mPAP) of 79 mmHg, pulmonary arterial wedge pressure (PAWP) of 11 mmHg, and pulmonary vascular resistance (PVR) of 18.3 Wood Units at diagnosis. The cardiac output and cardiac index were severely impaired with 2.96 l/min and 1.6 l/min/m^2^, respectively. She was in the WHO functional class III with a 6-min walking distance of 342 m and NT-proBNP level 1100 pg/ml and was treated with a triple combination therapy of sildenafil, macitentan, and inhaled iloprost.

The *SMAD5* variant c.1175T>C p.(Leu392Pro), also called p.L392P, leads to an exchange of the highly conserved amino acid leucin by proline at amino acid position 392 located in the MH2 protein binding domain of *SMAD5* in exon 7 of 8. The variant was predicted to be pathogenic by the *in silico* prediction program REVEL with a score of 0.97 (0.75–1.0 considered pathogenic). It was absent in >800000 probands (GnomAD v.4.1.0) including > 60000 healthy controls (GnomAD v.2.1.1).

### Patient with congenital heart disease associated PAH carried *SMAD5* missense variant c.277T>A p.(Trp93Arg)

The second variant in *SMAD5* c.277T>A p.(Trp93Arg), also called p.W93R, was found in a female patient with CHD-APAH. The patient had a closure of a ventricular septal defect at 2 years and developed PAH aged 29 years. The mPAP was 39 mmHg, PAWP 5 mmHg, PVR 9.9 Wood Units, cardiac output 3.5 l/min, and cardiac index 2.2 l/min/m^2^. The 6-min walking distance was 390 m. She was in WHO functional class III. The family history of the patient was negative for PAH, while the father of the index patient also had a ventricular septal defect and died aged 33 years of an unclear cause. The patient had neither siblings nor children (Figure 1B). She was treated with monotherapy of bosentan due to intolerance of further targeted PAH drugs.

The *SMAD5* variant p.W93R is located in exon 3 in the MH1 DNA binding domain of *SMAD5* leading to an exchange of the highly conserved amino acid tryptophan by arginine. The *in silico* prediction score indicated pathogenicity (REVEL 0.80). This variant was also absent in >800000 probands (GnomAD v.4.1.0) including >60000 healthy controls (GnomAD v.2.1.1).

Without further functional data, both *SMAD5* variants were classified as variants of uncertain significance (class III) according to the ACMG variant classification guidelines [15,20]. Thus, further experiments were required to explore their relevance for PAH pathology.

### Establishing cellular models using transient expression of c.1175T>C p.(Leu392Pro) and c.277T>A p.(Trp93Arg) in PASMCs

**Figure 2:**
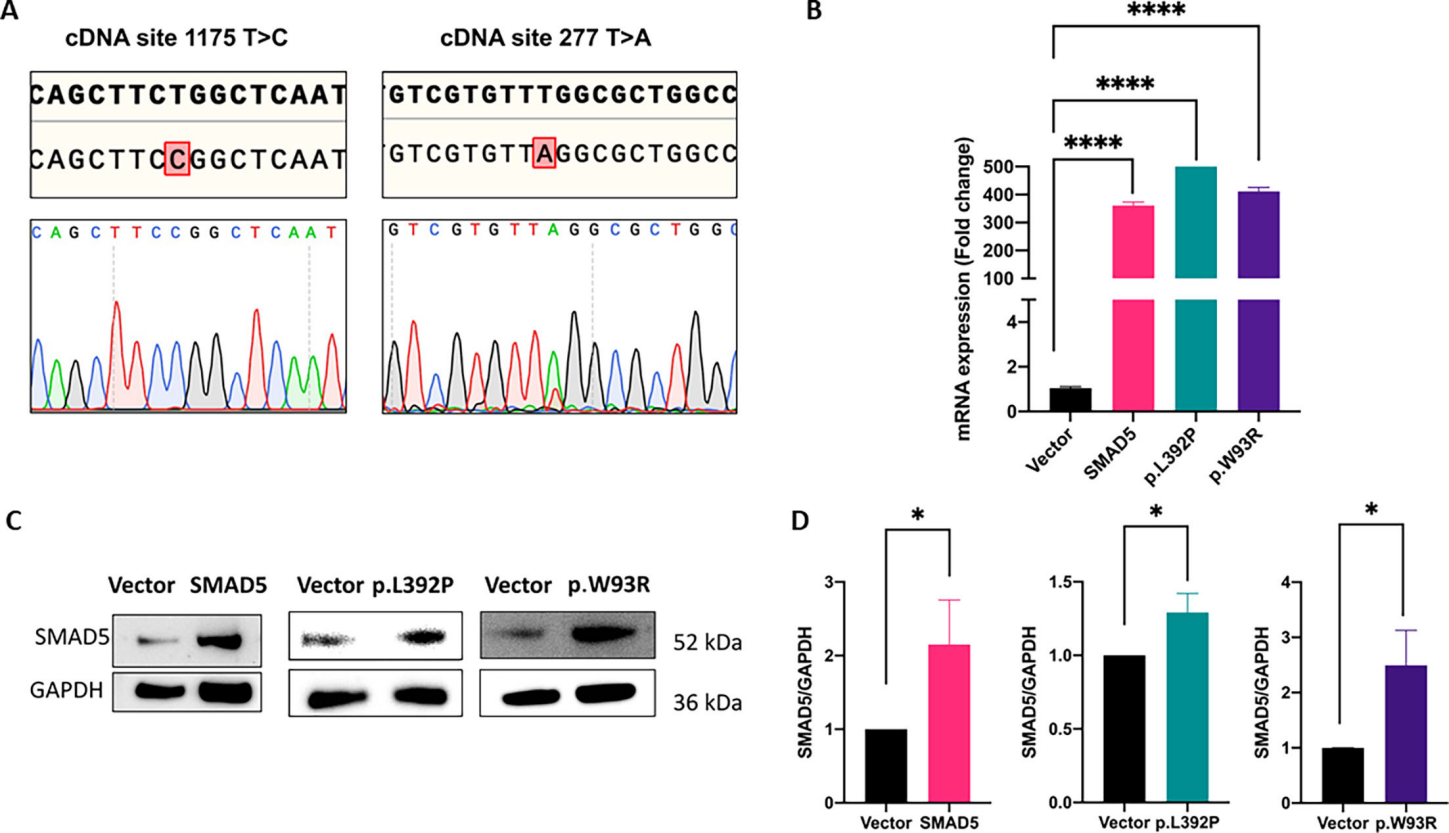
Validation of wt, c.1175T>C p.(Leu392Pro), and c.277T>A p.(Trp93Arg) expression in PASMCs. Cells were transfected with the empty plasmid pCMV6 (vector), plasmid with wt *SMAD5* (SMAD5), plasmid with *SMAD5* containing the variant p.L392P or variant p.W93R using Lipofectamine LTX. (**A**) RNA was extracted from transfected PASMCs, copy DNA generated, and *SMAD5* Sanger sequenced to confirm the presence of the respective missense variants. (**B**) Overexpression of *SMAD5* at mRNA level was confirmed by qPCR 24 h after transfection. *SMAD5* expression was normalized to *GAPDH* and subsequently to vector; *n* = 3 biological and *n* = 3 technical replicates. (**C**) Overexpression of respective SMAD5 proteins 48 h after transfection in comparison with the empty vector control was confirmed by Western blotting. (**D**) Quantification of the blots in C, calculated as the ratio of the density of SMAD5 band to GAPDH band; *n* = 3 biological replicates. *P<0.05; ****P<0.0001.

For a transient overexpression of both variants, they were each integrated in the SMAD5-pCMV6 plasmid system by site-directed mutagenesis and transfected into PASMCs (lot 1809196). Sanger sequencing confirmed the presence of both variants in cDNA from transfected PASMCs (Figure 2A). To measure differences in mRNA expression, we performed qPCR 24 h post-transfection with primers flanking exons 2 and 3 (Table S1). *SMAD5* wt, *SMAD5*-p.L392P, and *SMAD5*-p.W93R mRNA levels were significantly increased compared with cells transfected with the empty vector (Figure 2B). To test, whether the differences in the expression of *SMAD5*-p.L392P were also visible at the protein level, we performed Western blot for *SMAD5* in 48 h post-transfection. In comparison with cells transfected with the empty plasmid, total SMAD5 wt protein, *SMAD5*-p.W93R, and *SMAD5*-p.L392P protein were also significantly increased in three independent experiments (Figure 2C–D). Thus, our results confirmed successful overexpression of wt and mutated SMAD5 at mRNA and protein levels, demonstrating the utility of this system for further analyses.

### *SMAD5* c.1175T>C p.(Leu392Pro) showed increased cell viability, whereas c.277T>A p.(Trp93Arg) reduced cell viability

**Figure 3:**
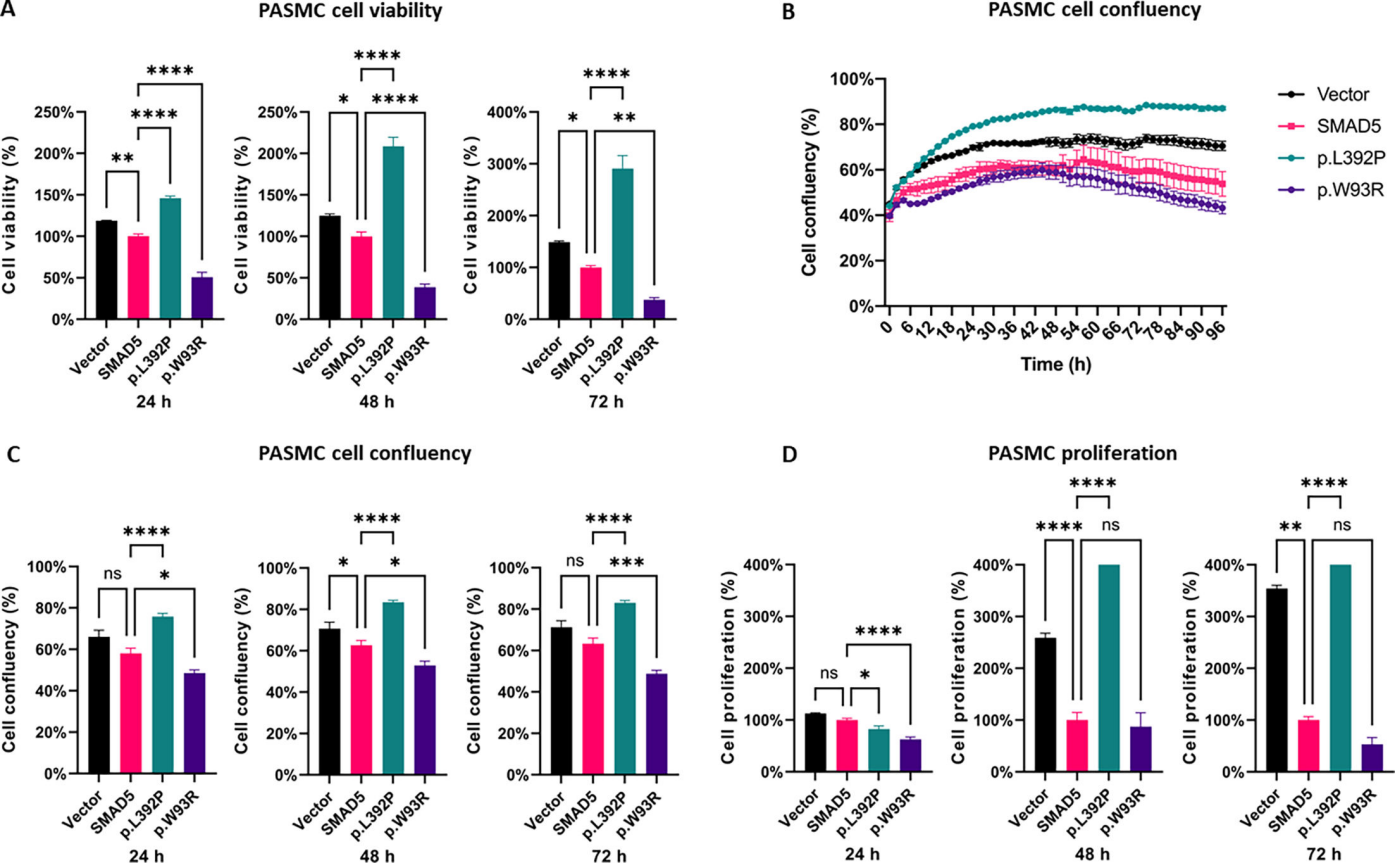
c.1175T>C p.(Leu392Pro) increased, and c.277T>A p.(Trp93Arg) decreased cell viability, confluency, and proliferation of PASMCs. PASMCs were transfected with the empty plasmid pCMV6 (vector), plasmid with wt *SMAD5* (*SMAD5*), plasmid with *SMAD5* containing variant p.L392P, or variant p.W93R using Lipofectamine LTX. (**A**) Cell viability was measured by cck-8 24 h, 48 h, and 72 h after transfection. OD was measured at 460 nm and normalized to wt *SMAD5*; *n* = 3 biological replicates. (**B**) Incucyte analysis of cell confluency from 0 h to 94 h after transfection with pictures taken every 2 h. (**C**) Quantification of cell confluency at three-time points; *n* = 3 biological replicates. (**D**) BrdU assay measured proliferation 24 h, 48 h, and 72 h after transfection. OD was measured at 450 nm and normalized to wt *SMAD5*; *n* = 3 biological replicates. *P<0.05; **P<0.01; ****P<0.0001; ns, non-significant.

Measuring cck-8 24 h, 48 h, and 72 h after transfection, we identified a significantly higher cell viability for *SMAD5*-p.L392P PASMCs (lot 1809196) compared with the wild type *SMAD5* (Figure 3A). In contrast, the *SMAD5*-p.W93R PASMCs showed significantly lower cell viability. The cell viability results were further confirmed by cell confluency measurements by the Incucyte Live Cell Analysis System as a second method (Figure 3B–C).

Of note, two different lots of PASMCs were tested during the experimental procedure. The initial cells used for cell viability experiments originated from a 45-year-old male African American donor with an unknown cause of death (lot 1792537). Results of *SMAD5*-p.L392P were consistent across the previous and the new donor cells (28-year-old Asian male with anoxia as cause of death, lot 1809196). However, the *SMAD5*-p.W93R variant in the former donor cells led to a significantly increased cell viability in the previous three independent cck-8 experiments (Supplementary Figure S2). This suggests not only the variant but also the genetic background of the donor may influence the outcome.

### Elevated proliferation may drive higher *SMAD5*-p.L392P cell viability and is reduced in *SMAD5*-p.W93R PASMCs

Cell viability is mainly driven by proliferation and apoptosis, which in turn may be influenced by genetic variants of relevant genes. To explore this relationship, we performed a BrdU assay to measure proliferation. At 48 h and 72 h after transfection, *SMAD5*-p.L392P cells (lot 1809196) showed a significantly augmented proliferation rate (Figure 3D) that may explain the increased cell viability of p.L392P cells. In contrast, the wt *SMAD5* cells and the p.W93R cells (lot 1809196) showed a significantly decreased proliferation rate (Figure 3D).

### Increased apoptosis may reduce cell viability in PASMCs with *SMAD5*-p.W93R but remains unchanged in *SMAD5*-p.L392P PASMCs

**Figure 4:**
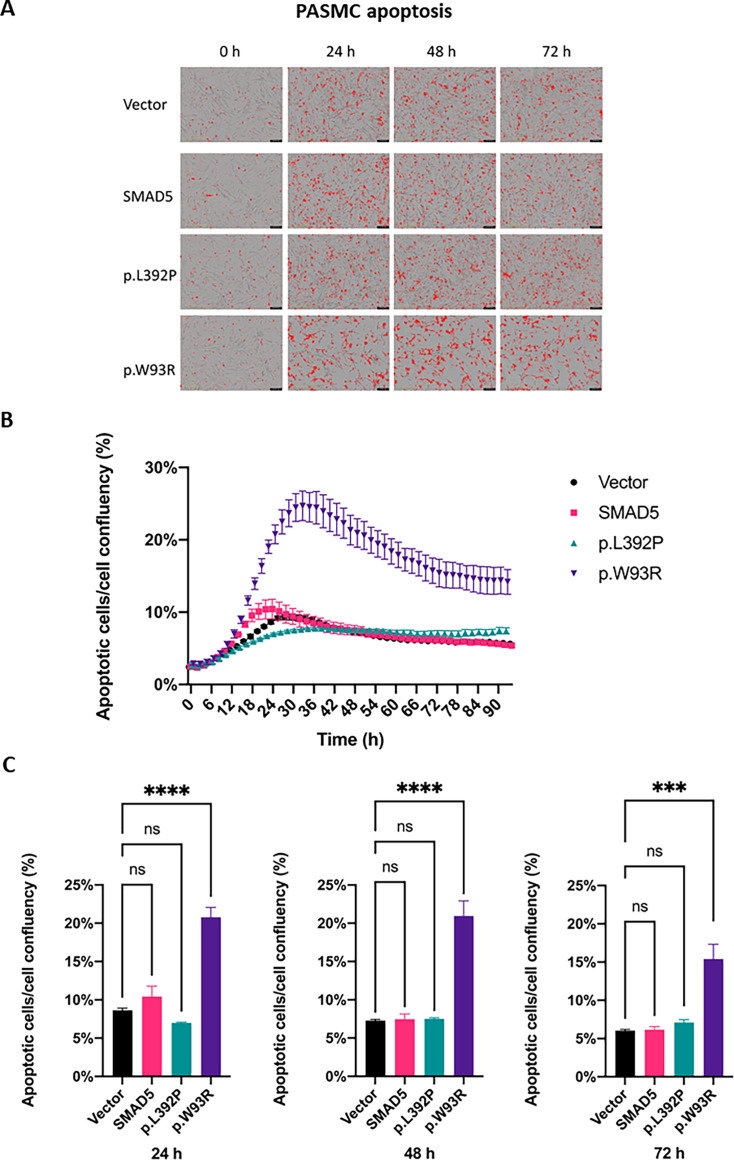
Apoptosis reduces cell viability of c.277T>A p.(Trp93Arg) but not of c.1175T>C p.(Leu392Pro) PASMCs. PASMCs were transfected with the empty plasmid pCMV6 (vector), plasmid with wt *SMAD5* (*SMAD5*), plasmid with *SMAD5* containing variant p.L392P, or variant p.W93R using Lipofectamine LTX. (**A**) Cell apoptosis was measured by Annexin V Red Dye using the Incucyte system at 0 h, 24 h, 48 h, and 72 h after transfection; magnification: 100 ×. Apoptotic cells are colored in red. (**B**) Incucyte analysis showing cell apoptosis relative to cell confluency from 0 h to 94 h after transfection; pictures were taken every 2 h. (**C**) Quantification of apoptosis relative to cell confluency. ***P<0.001; ****P<0.0001; ns, non-significant.

Apoptosis rate was measured by Annexin V Red Dye at different timepoints after transfection. No significant difference in apoptosis was observed between *SMAD5*-p.L392P PASMCs (lot 1809196), wt *SMAD5,* and empty vector plasmid (Figure 4). In contrast, apoptosis rate was significantly elevated for *SMAD5*-p.W93R PASMCs (lot 1809196) compared with wt *SMAD5* and empty vector plasmid (Figure 4), which may explain the decreased cell viability. These data strongly indicate that by each variant, different signaling pathways may be impaired, which may correlate with the position of the variant in different functional domains within the *SMAD5* gene.

### Reducing *SMAD5* expression has little effect on cell viability

Knowing the variant induced functional changes in PASMCs, we aimed to investigate whether a reduced expression of SMAD5 mimicking haploinsufficiency would have a different impact on cell viability. To this end, *SMAD5* was successfully knocked down with siRNA in PASMCs (lot 1809196) (Supplementary Figure S3 A-B). However, siSMAD5 treatment compared with unspecific siRNA-control treatment resulted in 72 h post targeting only in slightly reduced cell viability (Supplementary Figure S3). Since SMAD5 is part of the BMPR2 and ActR-IIA pathways, both were pharmacologically impaired to investigate their involvement in *SMAD5* variant induced cellular changes. A 24-h pharmacologic impairment of the canonical BMPR2-pathway (BMPR2-ALK1/2/3/6-SMAD1/5/8) by the ALK1/2/3/6 inhibitor LDN193189 or the ActR-IIA-pathway (ActR-IIA-ALK4/5/7-SMAD2/3) 24 h post-transfection could not ameliorate the highly increased cell viability of the *SMAD5*-p.L392P variant in PASMCs (lot 1809196) (Supplementary Figure S4), indicating the involvement of other pathways conferring the altered cell characteristics of the SMAD5 variants. The *SMAD5*-p.L392P mediated increased cell viability could, however, be rescued by a re-transfection of the cells with the wt *SMAD5* plasmid (Supplementary Figure S5) confirming the cell viability changes were due to the *SMAD5* variant.

Overall, we demonstrated that higher proliferation of PASMCs with the *SMAD5*-p.L392P variant compared with *SMAD5* wt led to an increase in cell viability *in vitro* in PASMCs. We conclude that it may be the same effect also *in vivo* and leading to narrowed pulmonary arteries and the development of PAH in the described HPAH family. We also showed that the *SMAD5*-p.W93R variant results, in contrast, in increased apoptosis and reduced cell viability of PASMCs. We assume that this leads to PAH in the CHD-APAH patient by a different mechanism than the *SMAD5*-p.L392P variant. Thus, both variants could be re-classified with the additional functional evidence as likely pathogenic.

## Discussion

This study is the first to report *SMAD5* as a novel gene for familial PAH. The c.1175T>C p.(Leu392Pro) variant was identified in a HPAH family and c.277T>A p.(Trp93Arg) in a CHD-APAH patient. Both variants were absent in >60000 controls and were predicted to be pathogenic by *in silico* prediction programs. PASMCs with the HPAH patient’s variant *SMAD5*-p.L392P showed a much greater cell viability due to an increased proliferation. Thus, the p.L392P variant could be a plausible cause of HPAH in the patient’s family leading to PASMC proliferation resulting in narrowed pulmonary arteries and a subsequent increase in pulmonary vascular resistance and pressure.

In contrast, the c.277T>A p.(Trp93Arg) *SMAD5* variant of the CHD-APAH patient resulted in significantly lower cell viability compared with wt. This phenotype was accompanied by a markedly increased apoptosis rate. Importantly, both variants showed opposite effects on cell viability, which significantly differ from wt *SMAD5* and, therefore, can be considered variants altering cell characteristics involved in the underlying molecular processes of PAH. Hence, we could re-classify both variants from variants of uncertain significance to likely pathogenic variants. Thus, we consider the *SMAD5* missense variant c.1175T>C p.(Leu392Pro) to be the disease-causing mutation for the HPAH family and the *SMAD5* c.277T>A p.(Trp93Arg) variant to be disease-causing for the CHD-APAH patient.

### SMAD5 in PAH

The alternative receptor SMAD, SMAD8, encoded by the gene *SMAD9* is a well-established PAH gene [6] with a functional overlap with *SMAD5* [21]. For *SMAD9,* 20 PAH-causing variants in the MH1 and MH2 domains have been described of which 19 are in fact missense variants (HGMD professional database v.2023.3). Interestingly, a family with a pathogenic *SMAD9* variant and a healthy carrier has been described [22]. It is known that pathogenic *BMPR2* variants have a reduced penetrance of about 30%. Hence, the healthy variant carrier of the likely pathogenic *SMAD5* in family 1 of our study suggests also an autosomal dominant inheritance with reduced penetrance for (likely) pathogenic *SMAD5* variants. While a variable expressivity is also known, e.g., for the age of onset of pathogenic *BMPR2* variant carriers, further *SMAD5* families remain to be described to characterize this genetic trait.

Homozygous knockout of *Smad5* in mice resulted in embryonic lethality [23]. A tissue-specific knockout of *SMAD5* in vascular smooth muscle and endothelial cells in mice led to a reduced exercise capacity and reduced left ventricular contractility in female mice in comparison with male knock-out mice and with control mice of both sexes with the *SMAD5* wt gene [10]. Unfortunately, the right heart and pulmonary artery pressures were not explicitly analyzed.

A recent study revealed an expression of quantitative trait loci in an intron of *SMAD5* which was associated with higher expression of *SMAD5* [24]. Moreover, the same study showed a reduced *SMAD5* mRNA expression in peripheral blood in 359 PAH patients compared with 72 age and sex-matched healthy controls. Rhodes and colleagues concluded that *SMAD5* is involved in PAH development, which is also supported by the data provided in this study.

### *SMAD5* variants in two different protein domains: divergent results

SMAD5 acts as a transcriptional regulator in processes such as embryonic development [25], cell differentiation [10], angiogenesis [10], and tissue homeostasis [11]. Its two functional domains, the MH1 and MH2 domains, are shared by all SMAD proteins from SMAD1 to SMAD9.

The *SMAD5*-p.L392P variant was located in the MH2 protein binding domain responsible for forming a homodimer with another SMAD5 protein or a hetero-trimer together with and an additional monomer of SMAD4 [26]. Missense mutations within the *SMAD2* MH2 domain reduced SMAD4 binding in comparison with wt proteins [27]. Thus, MH2 missense variants have the potential to disturb the protein’s core structure, impair the formation of stable heteromeric or homomeric SMAD complexes, hinder receptor-dependent R-SMAD phosphorylation, or cause instability in the SMAD proteins [28]. Therefore, MH2 domain mutations may affect tissue homeostasis altering proliferation as seen for the *SMAD5*-p.L392P variant.

Within the nucleus, the SMAD5 complex binds DNA with its MH1 domain. Target genes include the regulatory SMADs, SMAD6, and SMAD7, as well as other transcription factors such as ID1, ID2, and ID3, which are involved in regulation of proliferation and cell differentiation [29,30]. Moreover, more than 90% of cell cycle genes relevant to cellular growth are targeted by SMAD5 [31]. The *SMAD5*-p.W93R was located in the MH1 DNA binding domain. A functional study of a missense variant, which was identified in the MH1 DNA binding domain of *SMAD9* in a 7-year-old girl with severe PAH, revealed reduced DNA binding and impaired gene activation compared with wt SMAD9 [21]. Thus, processes regulated by downstream target genes are most likely altered such as apoptosis [30] as seen for the *SMAD5*-p.W93R variant.

In our study, cells exhibited significantly reduced (*SMAD5*-p.W93R) or increased (*SMAD5*-p.L392P) viability compared with wt SMAD5 depending on genetic background. This underscores the critical importance of maintaining the delicate balance of endogenous SMAD5 protein levels, as any deviation, whether in excess or deficiency, appears to impact cell viability. PASMCs in PAH are in general characterized by higher proliferation and lower apoptosis [32,33]. However, it was also shown that some disease-causing *BMPR2* mutations led to an increase in apoptosis [34] as we have seen for the *SMAD5*-p.W93R variant. Thus, it is most likely the disturbed balance of the TGF-β signaling in all PAH-relevant cell types resulting in the manifestation of PAH. In particular, the role of SMAD5 as a regulator of further transcription factors and cell cycle relevant genes might lead to a large effect by a single missense variant within a functional domain.

### Limitations

We were unable to obtain a sample from the father of the *SMAD5*-p.L392P index patient, while the mother had already died of PAH aged 42 years. Hence, there was not enough clinical data to perform a co-segregation of the variant with the disease. The 10-year-old index patient’s son was a healthy variant carrier up to now, but since the penetrance of PAH is incomplete and the mean age of the patients with idiopathic PAH or familial PAH in the contemporary registries from the Western world ranges from 45 to 65 years [35], a disease manifestation later in life cannot be excluded.

In our investigation, we exclusively employed *in vitro* experiments, limiting our ability to comprehensively assess the impact of the variants on pulmonary artery pressure in a living organism. Using *in vivo* experiments with established PH rodent models would allow for a more thorough evaluation of the underlying physiological changes and processes which could not be conclusively determined in our cell culture-based, variant over-expression model. Future research should also focus on the variant’s impact on cell cycle genes and altered regulatory networks of SMAD5 by, e.g., employing chromosome immune-precipitation sequencing. Moreover, the exact molecular mechanism of action of the two SMAD5 variants on the cellular phenotype could not be clarified. Further experiments investigating downstream signaling such as expression of key target genes and bone morphogenetic protein (BMP) responsive element reporter assays would have added important information and will be pursued in future studies. Furthermore, additional experiments to verify our functional findings with a different experimental technique would have strengthened our results.

Finally, it is essential to highlight that when working with primary donor cells, variations in donor health, ethnicity, and age backgrounds may lead to divergent experimental outcomes. The use of several sets of PASMCs of other donors, human-induced pluripotent stem cells (hiPSCs) from patients, or hiPSCs altered by CRISPR/Cas9 or *in vivo* models was beyond the scope of this study. The development of further human cellular models would enhance our understanding of *SMAD5* variants’ mechanism of action.

### Conclusion

In summary, we are the first to identify *SMAD5* as a novel gene for PAH. The HPAH patient’s variant p.L392P in *SMAD5* could increase cell viability conferred by an increase in cell proliferation. The CHD-APAH patient’s variant p.W93R showed a lower cell viability compared with wt SMAD5 accompanied by a markedly increased apoptosis rate. The mechanism of variant-induced increased or decreased viability remains to be established in detail. Due to the familial aggregation, clinical findings, and the functional evidence the variants could be classified as likely pathogenic. *SMAD5* remains to be validated as a novel PAH gene in further studies and other PAH patient cohorts.

**Clinical perspectives**

- Pulmonary arterial hypertension (PAH) is a rare disease with a potential genetic cause, which may remain unknown in many patients. Two rare missense variants were identified in PAH patients within the not yet established PAH gene *SMAD5*.
- Functional characterization of both detected *SMAD5* variants in pulmonary arterial smooth muscle cells was undertaken to be able to classify them as disease-causing.
- The present study suggests that *SMAD5* is a novel PAH gene that should be considered for genetic testing in PAH patients. Identified variants enable familial co-segregation and the identification of variant carriers at risk of PAH development to allow early diagnosis and treatment.

## Supplementary material

Supplementary Figures S1–S5

## Data Availability

All data generated and analyzed during the current study are included in this article or from the corresponding authors on reasonable request.

## Open Access

This article has been published open access under our Subscribe to Open programme, made possible through the support of our subscribing institutions, learn more here: https://portlandpress.com/pages/open_access_options_and_prices#conditional

## Abbreviations

ACMG: American College of Medical Genetics and Genomics
*BMPR2*: *bone morphogenetic protein receptor type 2*
BrdU: bromodeoxyuridine
CHD-APAH: PAH associated with congenital heart disease
CT: cycle threshold
GAPDH: glyceraldehyde 3-phosphate dehydrogenase
HPAH: heritable PAH
MH: MAD homology
NT-proBNP: N-terminal pro-brain natriuretic peptide
OD: optical density
PAH: pulmonary arterial hypertension
PASMCs: pulmonary artery smooth muscle cells
PAWP: pulmonary arterial wedge pressure
PBS: phosphate-buffered saline
PVR: pulmonary vascular resistance
REVEL: rare exome variant ensemble learner
TGF-β: transforming growth factor beta
VSD: ventricular septal defect
WHO: World Health Organization
cDNA: copy DNA
hiPSCs: human-induced pluripotent stem cells
mPAP: mean pulmonary arterial pressure
mRNA: messenger ribonucleic acid
qPCR: quantitative polymerase chain reaction
siRNA: Short interfering RNA
wt: wild-type
α-SMA: alpha-smooth muscle actin
