# Supplementary material for: SMAD5 as a novel gene for familial pulmonary arterial hypertension
Source: Clin Sci (Lond). 2025 Jan 15;139(1):15–27. doi: 10.1042/CS20241340 (PMC12204011; doi:10.1042/CS20241340)
Supplement: Supplementary Figures S1–S5 [file CS-139-01-CS20241340-s001.docx]

**Supplemental Material**

**Supplemental Figures**

**
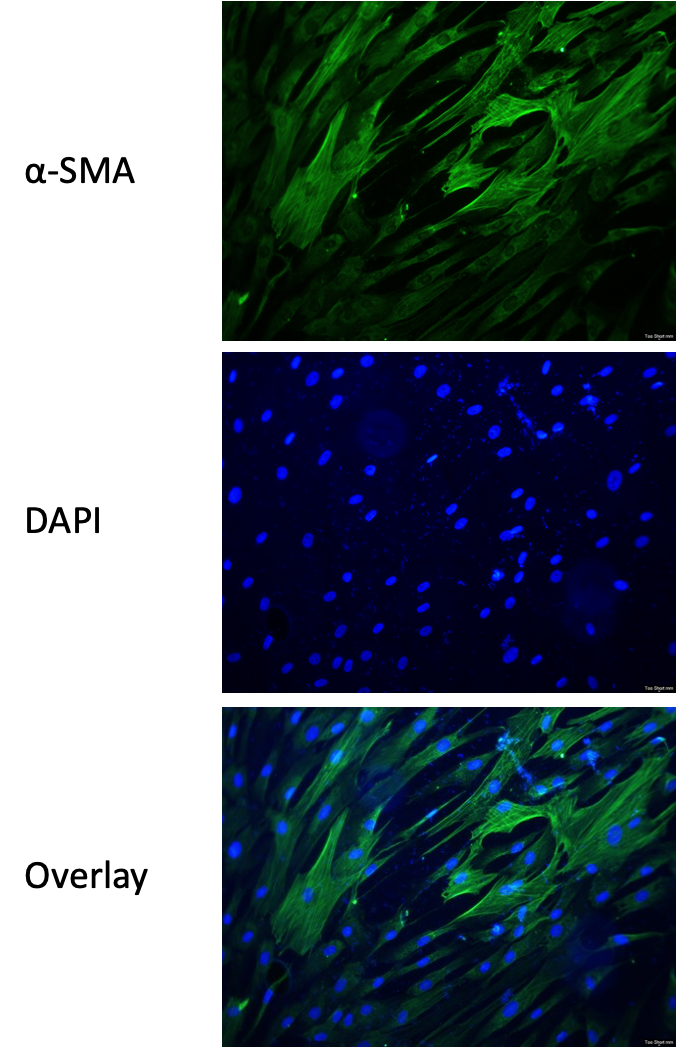
**

**Fig. S1 | Characterization of PASMCs.**

PASMCs treated with medium 231 + Smooth Muscle Differentiation Supplement (SMDS) for 4 days; alpha smooth muscle actin (α-SMA) positive cells are green; 4’,6-diamidino-2-phenylindole (DAPI) stains nuclei in blue. Magnification: 200 x; PASMCs: pulmonary artery smooth muscle cells (lot 1792537).

**
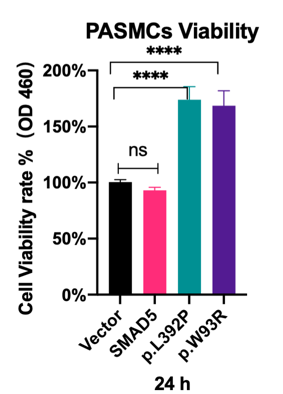
**

**Fig. S2 | Cell viability of lot 1792537 PAMSC.**

PASMCs were transfected with the empty plasmid pcmv6 (Vector), plasmid with wildtype *SMAD5* (*SMAD5*), plasmid with *SMAD5* variant p.L392P or variant p.W93R using lipofectamine LTX. (A) Cell viability measured by cck-8 24 h after transfection. Lot 1792537: PASMCs from a 45 year old African American male donor with unknown cause of death (ThermoFisher, USA). ****: p<0.0001; n.s.: non-significant; PASMC: pulmonary artery smooth muscle cells (lot 1792537).


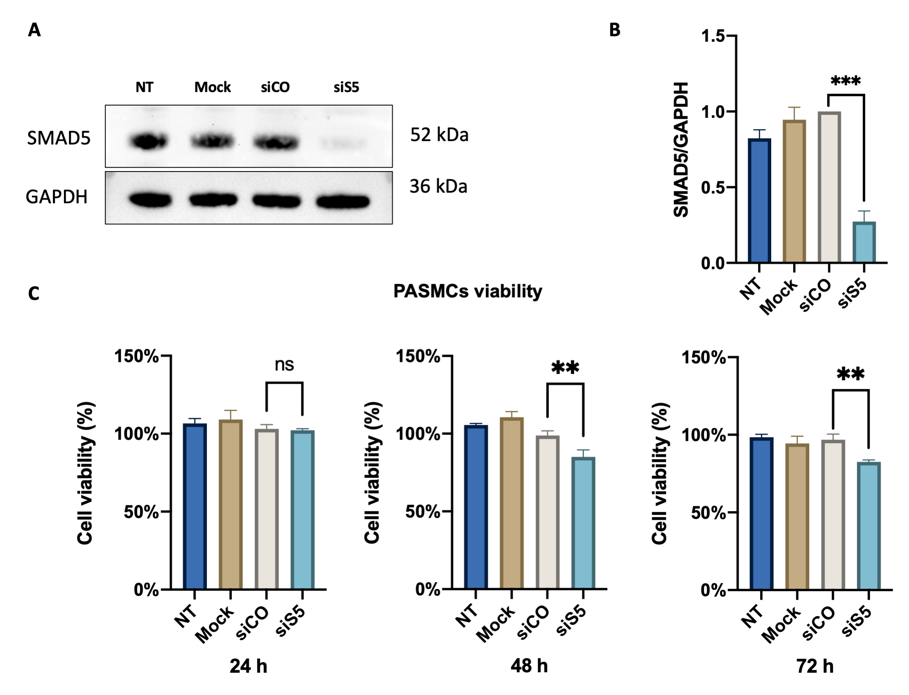


**Fig. S3 | Mild effect of siRNA *SMAD5* knockdown on cell viability.**(A) A 5-fold lower SMAD5 protein expression compared to unspecific siRNA-control treatment was observed. PASMCs were either not transfected (NT), treated only with lipofectamine 3000 (Mock), with siRNA-control (siCO) or siRNA-*SMAD5* (siS5). Protein lysates were collected 48 h after transfection and were immunoblotted for SMAD5 and GAPDH. (B) SMAD5 protein expression was normalized each to GAPDH. Fold change is relative to siCO. (C) Cell viability of siS5 transfected PASMCs measured by cck-8 24 h after transfection showed no difference to siCO and only a slight reduction after 48 h and 72 h. OD was measured at 460 nm and normalized to siCO. **: p<0.01; ***: p<0.001; n.s.: non-significant; PASMCs: pulmonary artery smooth muscle cells (lot 1809196).

**
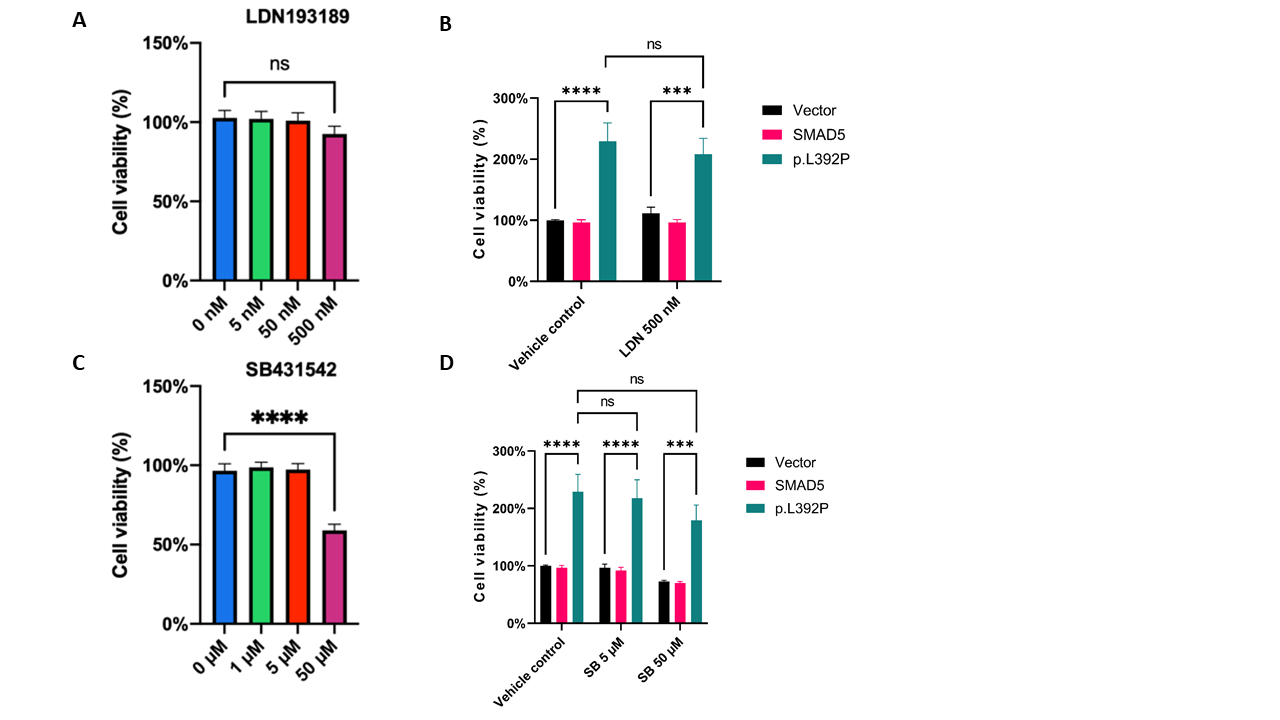
**

**Fig. S4 | Cell viability increase could not be impaired by inhibition of ALK1/2/3/6 or ALK4/5/7 in PASMCs.**(A) PASMCs were treated with 5 nM, 50 nM or 500 nM of LDN193189 (ALK1/2/3/6 inhibitor). Cell viability was measured after 24 h. OD was measured at 460 nm and normalized to cells without treatment (0 nM). (B) PASMCs were transfected with the empty plasmid (Vector), plasmid with wild type *SMAD5* or plasmid with *SMAD5* containing p.L392P using lipofectamine LTX. 24 h later cells were incubated with 500 nM ALK1/2/3/6 inhibitor LDN193189. Cell viability was measured after another 24 h. OD was measured at 460 nm and normalized to Vector treated with SMGS. (C) PASMCs were treated with 1 µM, 5 µM or 50 µM of ALK4/5/7 inhibitor SB431542. Cell viability was measured after another 24 h. OD was measured at 460 nm and normalized to cells without treatment (0 µM). The substantial reduction at the highest concentration of 50 µM could be due to off-target effects. (D) PASMCs were transfected with the empty plasmid (Vector), plasmid with *SMAD5* or plasmid with *SMAD5* containing p.L392P using lipofectamine LTX. 24 h later cells were incubated with 5 µM or 50 µM SB431542 and cell viability was measured after another 24 h by cck-8. OD was measured at 460 nm and normalized to Vector treated with SMGS. ***: p<0.001; ****: p<0.0001; ns: non-significant, PASMC: pulmonary artery smooth muscle cells (lot 1809196), SMGS: smooth muscle cell growth supplement medium.


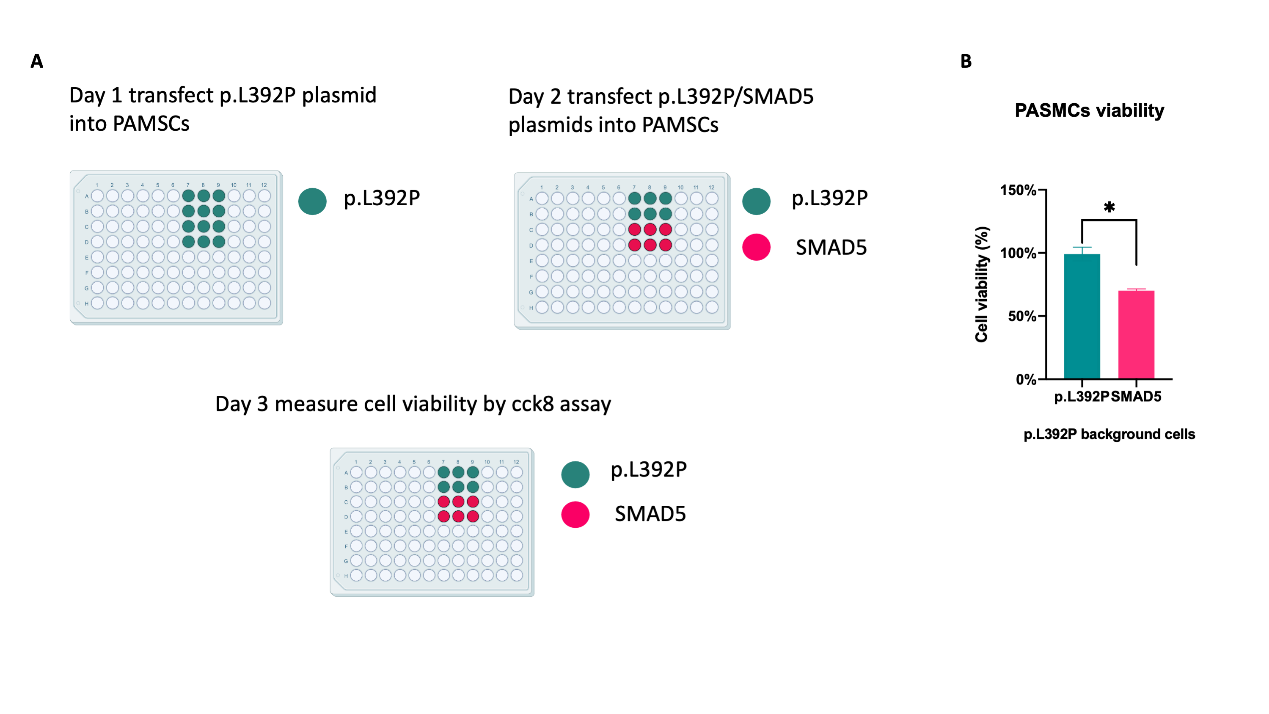


**Fig. S5 | Loss of c.1175T>C p.(Leu392Pro) lowered PASMCs viability.**The reversal of the phenotype (increased cell viability) could be achieved in PASMCs with the p.L392P variant by re-transfection with the wild type plasmid. (A) Overview of the transfection protocol. (B) Cell viability measured by cck8 assay. OD was measured at 460 nm and normalized to cells with two p.392P-*SMAD5* transfections. PASMCs: pulmonary artery smooth muscle cells (lot 1809196); *:p<0.05
